# Antiapoptotic Factor Humanin Is Expressed in Normal and Tumoral Pituitary Cells and Protects Them from TNF-α-Induced Apoptosis

**DOI:** 10.1371/journal.pone.0111548

**Published:** 2014-10-31

**Authors:** María Florencia Gottardo, Gabriela Jaita, María Laura Magri, Sandra Zárate, Mariela Moreno Ayala, Jimena Ferraris, Guadalupe Eijo, Daniel Pisera, Marianela Candolfi, Adriana Seilicovich

**Affiliations:** Instituto de Investigaciones Biomédicas, Universidad de Buenos Aires-CONICET, Buenos Aires, Argentina; University of Michigan School of Medicine, United States of America

## Abstract

Humanin (HN) is a 24-amino acid peptide with cytoprotective action in several cell types such as neurons and testicular germ cells. Rattin (HNr), a homologous peptide of HN expressed in several adult rat tissues, also has antiapoptotic action. In the present work, we demonstrated by immunocytochemical analysis and flow cytometry the expression of HNr in the anterior pituitary of female and male adult rats as well as in pituitary tumor GH3 cells. HNr was localized in lactotropes and somatotropes. The expression of HNr was lower in females than in males, and was inhibited by estrogens in pituitary cells from both ovariectomized female and orquidectomized male rats. However, the expression of HNr in pituitary tumor cells was not regulated by estrogens. We also evaluated HN action on the proapoptotic effect of TNF-α in anterior pituitary cells assessed by the TUNEL method. HN (5 µM) *per se* did not modify basal apoptosis of anterior pituitary cells but completely blocked the proapoptotic effect of TNF-α in total anterior pituitary cells, lactotropes and somatotropes from both female and male rats. Also, HN inhibited the apoptotic effect of TNF-α on pituitary tumor cells. In summary, our results demonstrate that HNr is present in the anterior pituitary gland, its expression showing sexual dimorphism, which suggests that gonadal steroids may be involved in the regulation of HNr expression in this gland. Antiapoptotic action of HN in anterior pituitary cells suggests that this peptide could be involved in the homeostasis of this gland. HNr is present and functional in GH3 cells, but it lacks regulation by estrogens, suggesting that HN could participate in the pathogenesis of pituitary tumors.

## Introduction

Humanin (HN) is a 24-amino acid peptide originally isolated from a cDNA library of surviving neurons of familial Alzheimer’s disease [1]. The homologous rat gene encodes a 38 aminoacid peptide referred to as rattin (HNr). HN and HNr share aminoacid residues essential for homodimerization, secretion and neuroprotection [2], [3], [4]. The origin of HN family peptides is controversial, hypotheses being that they may be encoded in either the mitochondrial or nuclear DNA [4], [5], [6]. HN is expressed in the endothelial cell layer of human arteries and veins [7] and in carotid atherosclerotic plaques [8]. HN has also been detected in human skeletal muscle [9], plasma [10], testis and sperm [11]. HNr was shown to be expressed in a number of adult rat tissues such as cerebral cortex, hippocampus, heart, testis and uterus, among others [12]. So far, HNr expression in the rat pituitary has not yet been explored.

The regulatory mechanisms of HN biosynthesis remain largely unknown. Interestingly, HN concentration in plasma of both humans and mice as well as HNr concentration in the rat hypothalamus were shown to decrease with age [7], [13]. However, in a developmental expression pattern of HNr observed in rat testis, HNr increased with age [14]. Also, HN release from neurons increases when the cytoplasmic levels of cAMP and calcium rise [15]. In addition, insulin-like growth factor-binding protein-3 (IGFBP-3) interacts with HN, regulating its circulating levels [6], [16], [17], [18].

Many studies have demonstrated that HN suppresses cell death induced by various Alzheimer disease-related insults [15] and by some other injuries including serum deprivation [4]. The cytoprotective effects of HN have been described in different cell types including neurons [1], lymphocytes [19], pancreatic β cells [20], testicular germ cells and Leydig cells [14], [16], [20].

Rate of proliferation and cell death control the number of cells in tissues. Alterations in signaling pathways that regulate apoptosis are involved in various pathological processes, including tumor development. In the anterior pituitary, lactotropes and somatotropes exhibit the highest rate of cell renewal followed by corticotropes, gonadotropes and thyrotropes [21]. In male rats, approximately 1.5% of anterior pituitary cells proliferate every 24 h, while the percentage of proliferating cells in female rats doubles that in males. In rodents, the anterior pituitary undergoes proliferation and apoptosis processes during the estrous cycle that are associated with cyclic changes in the circulating levels of gonadal steroids [21]. In lactotropes and somatotropes, apoptosis is estrogen-dependent and occurs predominantly at proestrus, when circulating estrogen levels are the highest [22]. Estrogen induces a rapid apoptotic response in these cells by binding to estrogen receptor-α associated with the plasma membrane [23].

The aim of the present study was to evaluate the expression and action of HN peptides in anterior pituitary cells. We explored HNr expression in the anterior pituitary of female and male rats and in the somatolactotrope GH3 cell line, as well as modulation of this expression by gonadal steroids. Considering that survival and apoptosis of pituitary cells are regulated by the hormonal milieu, we evaluated the effect of HN on TNF-α-induced apoptosis of anterior pituitary cells from both gonadectomized female and male rats incubated in the presence of gonadal steroids.

Our results indicate that HN exerted antiapoptotic action on anterior pituitary cells from female and male rats and that gonadal steroids regulated HNr expression, suggesting that HN could be involved in tissue homeostasis in this gland. Our data also show that HN protected GH3 cells from apoptosis whereas HNr expression in these pituitary tumor cells was unresponsive to estrogen modulation.

## Materials and Methods

### Ethics Statement

Adult Wistar female and male rats were kept in accordance with the National Institutes of Health Guide for the Care and Use of Laboratory Animals. Rats were gonadectomized under ketamine (100 mg/Kg, i.p.) and xylazine (10 mg/Kg, i.p.) anesthesia and ketoprofen (5 mg/kg) for analgesia. Animals were euthanized by decapitation. Animal protocols were previously approved by the Ethics Committee of the School of Medicine, University of Buenos Aires (Res. N° 2742/2013).

### Drugs

All drugs and reagents were obtained from Sigma Chemical Co. (St. Louis, MO) except for phenol red-free Dulbecco’s modified Eagle’s medium and supplements (GIBCO, Invitrogen, Carlsbad, CA), FBS (GBO, Buenos Aires, Argentina), all terminal deoxynucleotidyltransferase-mediated dUTP nick end-labeling (TUNEL) reagents (Roche Molecular Biochemicals, Mannheim, Germany), antibodies against anterior pituitary hormones (Dr. A. Parlow, National Hormone and Pituitary Program, Torrance, CA), anti-guinea pig rhodamine-conjugated secondary antibody (Chemicon International, Temecula, CA), anti-rabbit IgG fluorescein-conjugated secondary antibody (Vector Laboratories, Burlingame, CA), anti-guinea pig Alexa 555-conjugated antibody (Invitrogen, Carlsbad, CA), Humanin peptide (Genemed Synthesis, Inc, San Antonio, TX) and the materials indicated below.

### Animals

Adult Wistar female and male rats (200–250 g) were kept in controlled conditions of light (12∶12 h light-dark cycles) and temperature (20–22°C). Rats were fed standard laboratory chow and water ad libitum. Rats were ovariectomized (OVX), orquidectomized (GNX) or sham-operated two weeks before experiments. Anterior pituitary glands were removed within minutes after decapitation.

### Cell culture

A pool of anterior pituitary cells from 2–4 OVX or GNX rats was used for each culture. Anterior pituitary glands were washed several times with Dulbecco’s Modified Eagle’s Medium (D-MEM) supplemented with 10 µl/ml MEM amino acids, 2 mM glutamine, 5.6 µg/ml amphotericin B, 100 µg/ml streptomycin (DMEM-S) and 3 mg/ml bovine serum albumin (BSA). Then, glands were cut into small fragments. Sliced fragments were dispersed enzymatically by successive incubations in DMEM-S-BSA, containing 0.75% trypsin, 10% fetal bovine serum (FBS) previously treated with 0.025% dextran-0.25% charcoal (FBS-DCC) to remove steroids and 45 U/µl deoxyribonuclease type I (Invitrogen). Finally, cells were dispersed by extrusion through a Pasteur pipette in Krebs buffer without Ca^2+^ and Mg^2+^. Dispersed cells were washed and resuspended in DMEM-S with 10% FBS-DCC. GH3 cells were cultured in flasks containing DMEM supplemented with 10 µl/ml MEM amino acids, 2 mM glutamine, 0.56 µg/ml amphotericin B, 100 U/ml penicillin, 100 µg/ml streptomycin, 5% FBS and 5% fetal horse serum. GH3 cells were harvested with 0.025% tripsin-EDTA. Cell viability assessed by trypan blue exclusion was over 90%. Anterior pituitary cells or GH3 cells were seeded on cover slides in 24-well tissue culture plates (1×10^5^ cells·ml^−1^·well^−1^) for TUNEL assay or in 24-well tissue culture plates (3×10^5^ cells·ml^−1^·well^−1^) for flow cytometry [fluorescence-activated cell sorter (FACS)] analysis. Cells were cultured for 24 h in DMEM-S with 10% FBS-DCC and then incubated for 24 h in phenol red-free, serum-free DMEM-S supplemented with 0.1% BSA (DMEM-S-BSA 0.1%) containing 17β-estradiol (E_2_, 10^−9^ M) alone or with 10^−7^ M ICI 182 780 (Tocris, Ellisville, MO), dihydrotestosterone (DHT, 10^−8^ M) or the corresponding vehicle (ethanol 1 or 10 µl/l). To determine apoptosis, cells were further preincubated with HN (2.5–10 µM) for 2 h before adding TNF-α (50 ng/ml) for 24 h in the same medium with or without E_2_ or DHT.

### Immunolocalization of HNr

The presence of HNr in pituitary sections and dispersed anterior pituitary cells was evaluated by indirect immunofluorescent staining. To obtain pituitary sections, anesthetized OVX rats were perfused through the left ventricle with cold 4% paraformaldehyde (PFA) in 0.1 M phosphate buffer, pH 7.4. The glands were quickly removed and kept in the same solution for 3 h at 4°C, then in 70% ice-cold ethanol for 24 h and embedded in paraffin. Pituitary sections (4 µm) were deparaffinized in xylene and rehydrated in graded ethanol. Dispersed pituitary cells seeded on glass coverslips were fixed in 4% PFA. Sections and cells were incubated for 1 h with anti-HNr antibody (Sigma, 1∶100), washed and incubated for 1 h with anti-rabbit IgG-fluorescein (Vector Laboratories, 1∶50). To identify lactotropes and somatotropes, cells were incubated for 1 h with guinea pig anti-rat prolactin (PRL) antiserum (1∶2500) or guinea pig anti-rat growth hormone (GH) antiserum (1∶1500) and then with anti-guinea pig Alexa 555-conjugated antibody (1∶100) for 1 h. Finally, slides were mounted with mounting medium for fluorescence (Vectashield, Vector Laboratories) containing 4′,6-diamidino-2-phenylindole, DAPI). Control slides were incubated with the corresponding normal serum or IgG subtype instead of primary antibody. Cells were visualized in a fluorescence light microscope (Axiophot, Carl Zeiss, Jena, Germany).

### Expression of HNr by flow cytometry

Cultured GH3 or anterior pituitary cells from OVX and GNX rats as well as dispersed pituitary cells from male and female rats were harvested with 0.025% trypsin-EDTA and washed in cold PBS. Cells were fixed with 0.1% PFA and then permeabilized with 0.1% saponin (MP Biomedicals, Solon, OH) for 10 min in the dark at RT. Next, cells were incubated with rabbit HNr antibody (1 µg/µl, Sigma) in PBS-0.05% saponin for 1 h at 37°C. Then, cells were washed and incubated with an anti-rabbit IgG antibody conjugated with fluorescein (1∶50, Vector Laboratories) in PBS-0.05% saponin for another 1 h at 37°C. Finally, cells were washed, resuspended in PBS and analyzed by flow cytometry (FACS) using a FACScan (Becton Dickinson, NJ, USA). Data analysis was performed using WinMDI 98 software. Cells were incubated with isotype control instead of primary antibody to determine the cut-off for HNr fluorescence.

### Microscopic detection of DNA fragmentation by TUNEL

After incubation with HN, cells were fixed in 4% PFA in PBS and permeabilized by microwave irradiation. DNA strand breaks were labeled with digoxigenin-dUTP using terminal deoxynucleotidyl transferase (Roche Molecular Biochemicals). To identify lactotropes and somatotropes, cells were incubated for 1 h with guinea pig anti-rat PRL antiserum (1∶2500) or guinea pig anti-rat GH antiserum (1∶1500). Next, slides were incubated with antidigoxigenin-fluorescein-conjugated antibody (1∶10) to detect incorporation of nucleotides in the 3′-OH end of damaged DNA and rhodamine-conjugated antiguinea pig secondary antibody (1∶200) in the same buffer. Slides were mounted with mounting medium for fluorescence (Vectashield with DAPI) for DNA staining and visualized in a fluorescence light microscope. The percentage of apoptotic anterior pituitary cells was calculated as [(TUNEL^+^)/total cells]×100, the percentage of apoptotic lactotropes as [(TUNEL^+^ PRL^+^)/total PRL^+^ cells]×100, and the percentage of apoptotic somatotropes as [(TUNEL^+^ GH^+^)/total GH^+^ cells]×100.

### Statistical analysis

The number of apoptotic cells evaluated by TUNEL was analyzed in duplicate slides from at least two independent experiments. Total cell number in each coverslip was evaluated by DAPI nuclear staining. Results were expressed as the percentage of TUNEL-positive cells, lactotropes or somatotropes ±95% confidence limits (CL) of the total number of cells counted in each specific condition. Differences between proportions were analyzed by the χ^2^ test. Differences in the percentage of HNr-positive cells and the mean of HNr fluorescence intensity per cell (Gmean) between groups was analyzed by Student’s t test or two-way ANOVA followed by planned comparisons. P<0.05 was considered significant. All experiments were performed at least twice.

## Results

### Pattern of HNr expression in anterior pituitary cells from male and female rats

**Figure 1:**
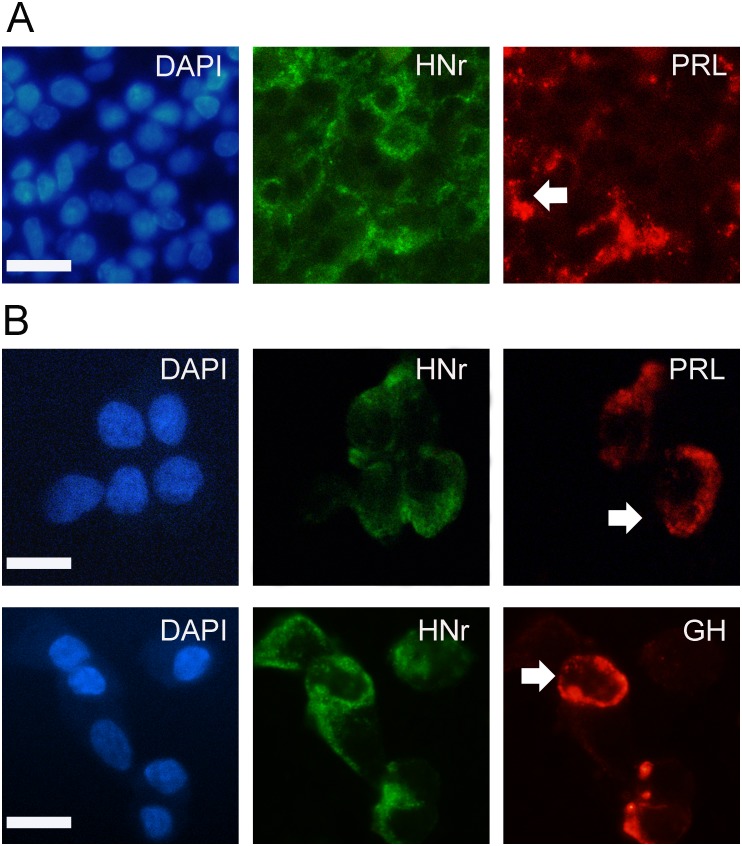
Expression of HNr in the anterior pituitary gland. (A) Anterior pituitary sections and (B) cultured anterior pituitary cells from OVX rats were processed for identification of HNr and pituitary hormones by double immunofluorescence. Left panels: nuclear staining with DAPI; middle panels: immunocytochemistry for HNr; right panels: immunocytochemistry for prolactin (PRL) or growth hormone (GH). Arrows show a lactotrope and a somatotrope expressing HNr. Scale bars: 10 µm.

**Figure 2:**
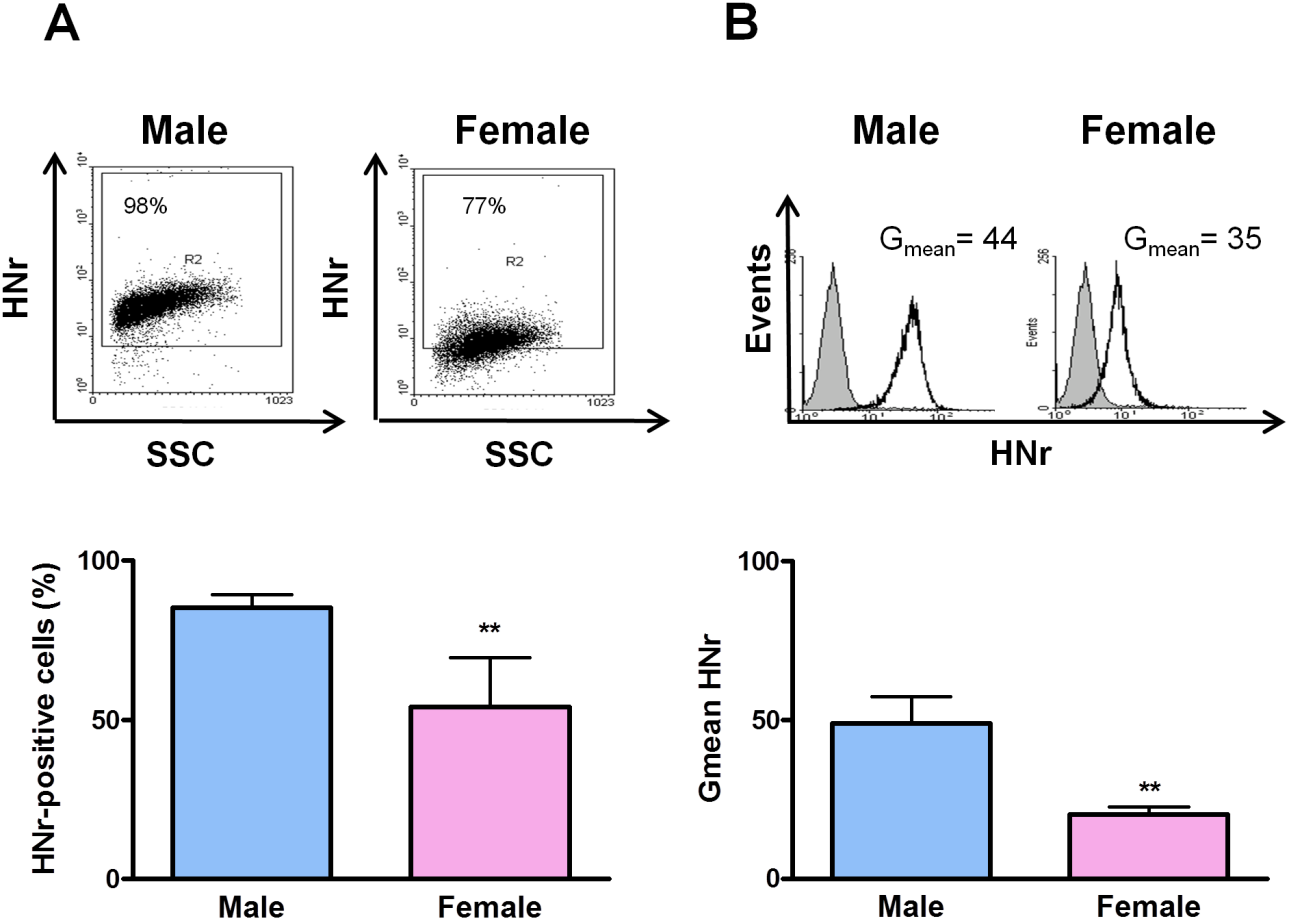
Anterior pituitary HNr expression in female rats is lower than in males. Dispersed anterior pituitary cells from intact male and female rats were immunostained for HNr and analyzed by flow cytometry. Each column represents the mean ± SE (n = 4 animals per group) of (A) the percentage of HNr-positive cells, and (B) the fluorescence intensity of HNr staining (Gmean). The upper panels show representative dot plots and histograms of HNr expression in anterior pituitary cells from male and female rats. **p<0.01, Student’s *t* test.

We first explored the expression of HNr in rat pituitary gland sections and primary culture by immunocytochemistry. HNr was present in the cytoplasm of a large number of anterior pituitary cells, localized in lactotropes and somatotropes (Fig. 1). We then compared rates of HNr expression in male and female anterior pituitary cells. As determined by FACS, both the percentage of anterior pituitary cells expressing HNr and the mean of HNr fluorescence intensity per cell (Gmean) was lower in females than in males (Fig. 2).

### Estrogens decreased HNr expression in anterior pituitary cells

**Figure 3:**
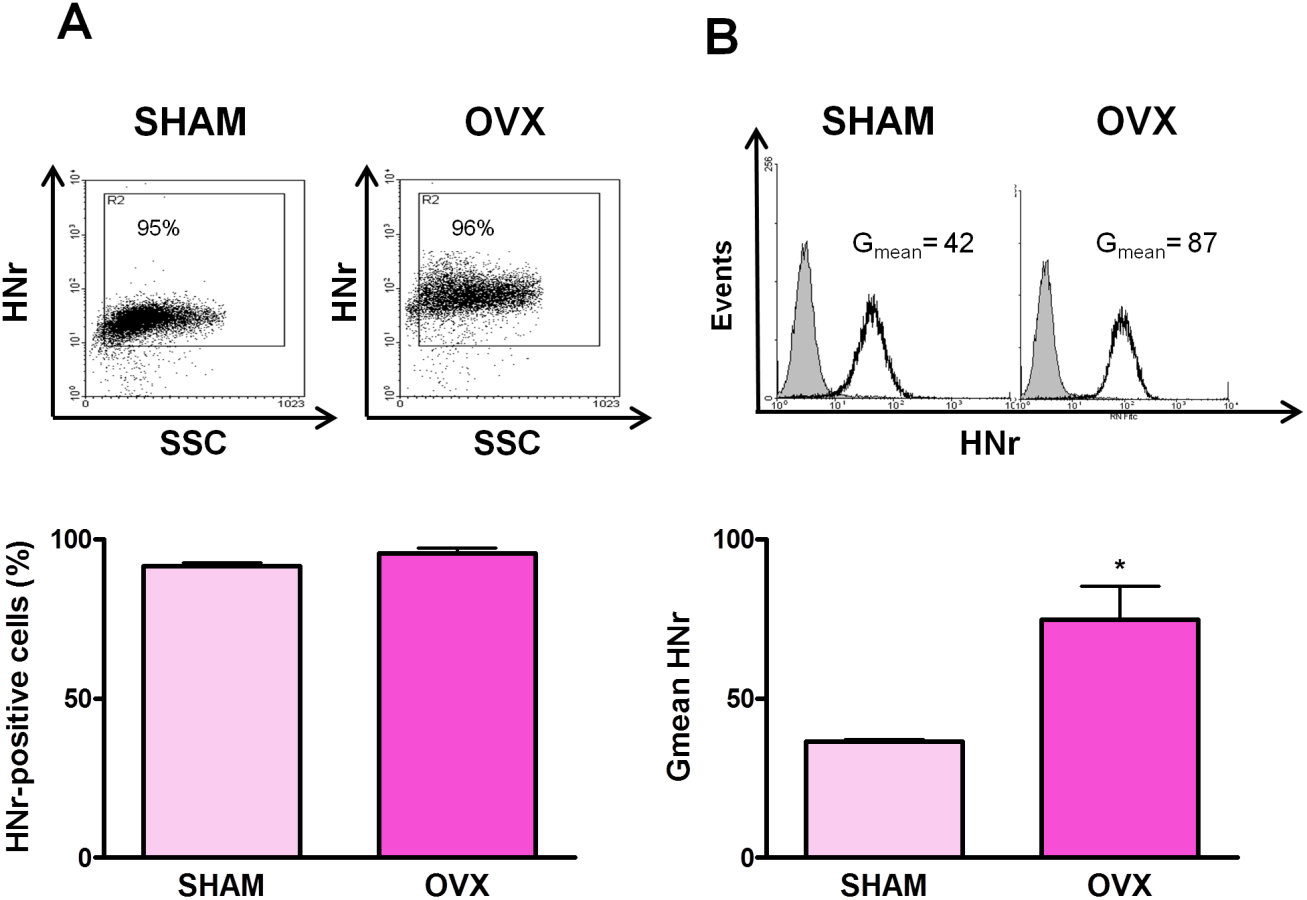
Ovariectomy increases anterior pituitary HNr expression. Dispersed anterior pituitary cells from SHAM and OVX rats were immunostained for HNr and analyzed by flow cytometry. Each column represents the mean ± SE (n = 4 animals per group) of (A) the percentage of HNr-positive cells, and (B) the fluorescence intensity of HNr staining (Gmean). The upper panels show representative dot plots and histograms of HNr expression in anterior pituitary cells from SHAM and OVX rats. *p<0.05, Student’s *t* test.

**Figure 4:**
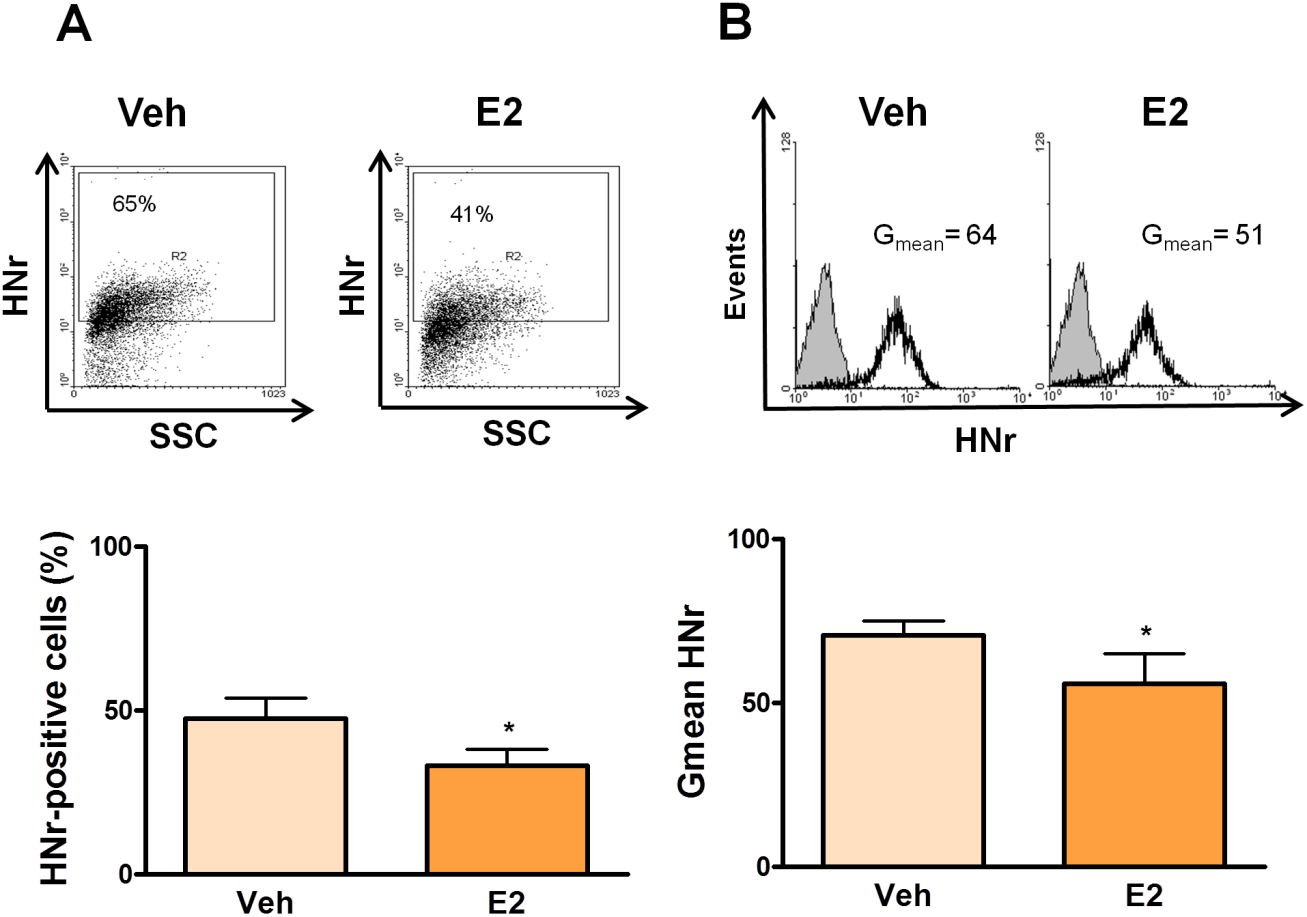
Estradiol decreases HNr expression in anterior pituitary cells from OVX female rats. Cultured anterior pituitary cells from OVX rats were incubated with 17β-estradiol (E_2_, 10^−9^ M) or vehicle (Veh, ethanol 1 µl/l) for 24 h, immunostained for HNr and analyzed by flow cytometry. Each column represents the mean ± SE of (A) the percentage of HNr-positive cells, and (B) the fluorescence intensity of HNr staining (Gmean) from 3 independent experiments (3 replicates each one). The upper panels show representative dot plots and histograms of HNr expression in anterior pituitary cells from OVX rats incubated with 17β-estradiol. *p<0.05, Student’s *t* test.

**Figure 5:**
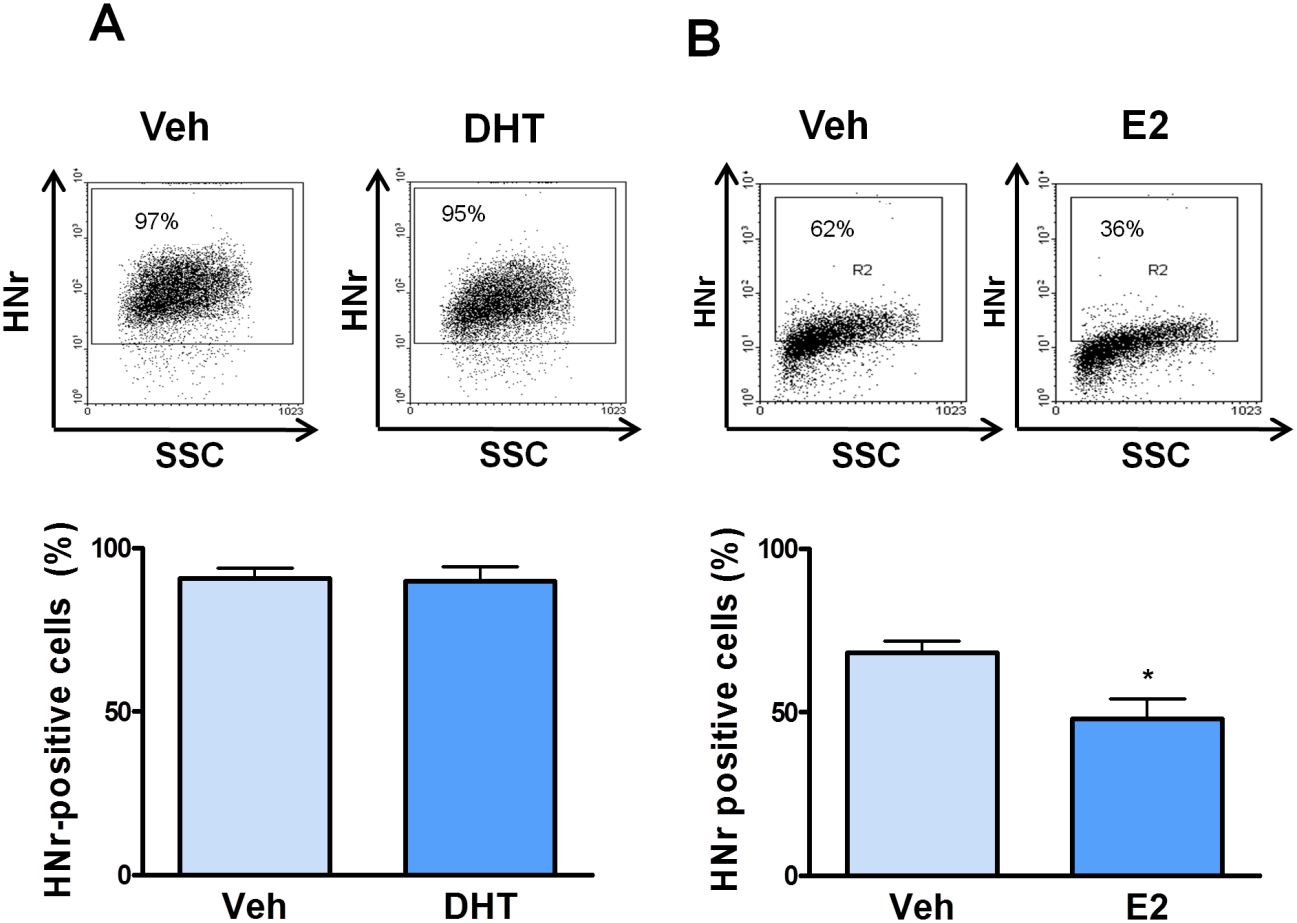
Estradiol decreases HNr expression in anterior pituitary cells from GNX male rats. Cultured anterior pituitary cells from GNX male rats were incubated with (A) dihydrotestosterone (DHT, 10^−8^ M) or vehicle (Veh, ethanol 10 µl/l) or (B) 17β-estradiol (E_2_, 10^−9^ M) or vehicle (Veh, ethanol 1 µl/l) for 24 h, immunostained for HNr and analyzed by flow cytometry. Each column represents the mean ± SE of the percentage of HNr-positive cells from 3 independent experiments (3 replicates each one). The upper panels show representative dot plots of HNr expression in anterior pituitary cells from GNX rats incubated with dihydrotestosterone or 17β-estradiol. *p<0.05, Student’s *t* test.

To evaluate whether estrogens regulate the expression of HNr in the female pituitary, we assessed by FACS HNr expression in anterior pituitary cells from SHAM-operated and OVX rats. Although ovariectomy did not modify the number of HNr-positive anterior pituitary cells, it increased HNr Gmean in these cells (Fig. 3), suggesting that gonadal hormones decrease HNr expression in anterior pituitary cells. To explore this possibility, anterior pituitary cells from OVX rats were incubated with 17β-estradiol (10^−9^ M). Estrogens decreased the percentage of HNr-positive cells and HNr Gmean in anterior pituitary cells (Fig. 4). The inhibitory effect of estrogens on HNr expression was not observed in the presence of the pure antagonist of estrogen receptors, ICI 182 780 (Fig. S1). However, in male rats, gonadectomy did not affect HNr expression in the anterior pituitary (Fig. S2). While DHT (10^−8^ M) had no effect on HNr expression in anterior pituitary cells from GNX rats, E_2_ inhibited its expression as we observed in female anterior pituitary cells (Fig. 5).

### HN blocked TNF-α-induced apoptosis of anterior pituitary cells

**Figure 6:**
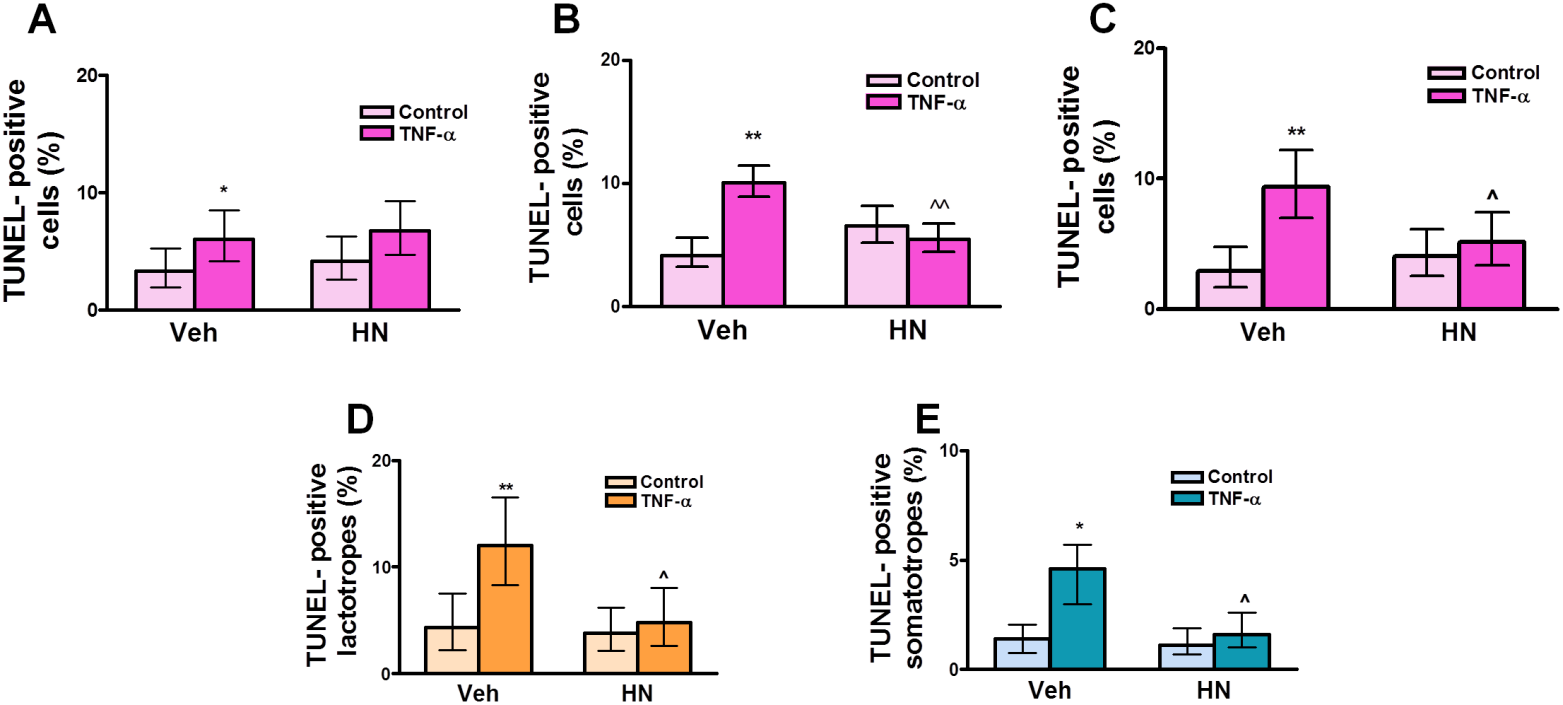
HN protects anterior pituitary cells from TNF-α-induced apoptosis in female rats. Cultured anterior pituitary cells from OVX rats were incubated with 17β-estradiol (E_2_, 10^−9^ M) for 24 h and then with HN 2.5 µM (A), 5 µM (B), or 10 µM (C) for 2 h before adding TNF-α (50 ng/ml) for an additional 24 h. Apoptosis was assessed by the TUNEL method. In cells incubated with 5 µM HN, apoptotic lactotropes (D) and somatotropes (E) were identified by immunofluorescence. Each column represents the percentage ± CL of TUNEL-positive anterior pituitary cells (A, B, C, n≥1500 cells/group), lactotropes (D, n≥1600 cells/group) or somatotropes (E, n≥1800 cells/group). Data from at least two separate experiments were analyzed by χ^2^ test. *p<0.05, **p<0.01 vs respective control without TNF-α; ∧p<0.05, ∧∧p<0.01 vs respective control without HN.

**Figure 7:**
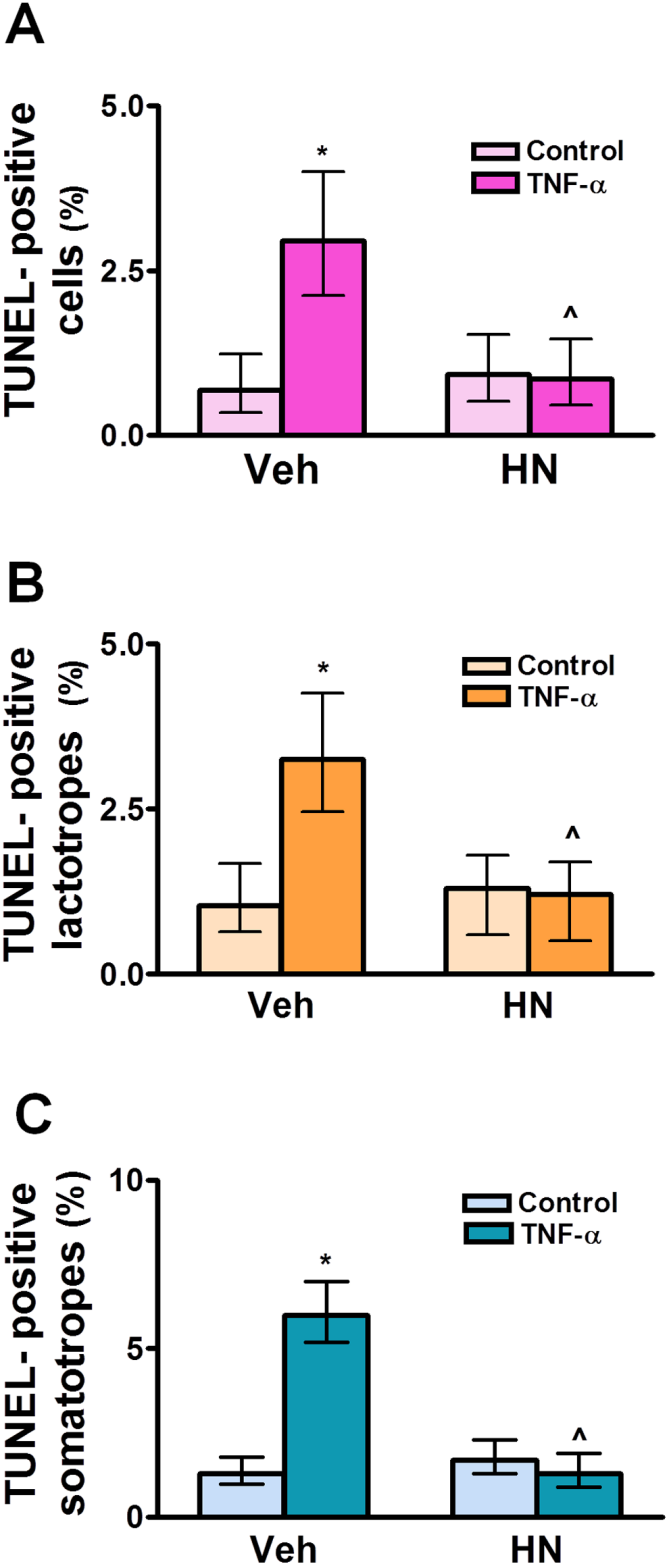
HN protects anterior pituitary cells from TNF-α-induced apoptosis in male rats. Cultured anterior pituitary cells from GNX male rats were incubated with dihydrotestosterone (DHT, 10^−8^ M) for 24 h and then with HN (5 µM) for 2 h before adding TNF-α (50 ng/ml) for an additional 24 h. Apoptosis was assessed by the TUNEL method and lactotropes and somatotropes detected by immunofluorescence. Each column represents the percentage ± CL of TUNEL-positive anterior pituitary cells (A, n≥1400 cells/group), lactotropes (B, n≥1400 cells/group) or somatotropes (C, n≥1300 cells/group). Data from at least two separate experiments were analyzed by χ^2^ test. *p<0.05 respective control without TNF-α; ∧p<0.05 vs respective control without HN.

We have previously reported that TNF-α induces apoptosis of anterior pituitary cells, especially lactotropes and somatotropes, in an estrogen-dependent manner [24], [25]. Since HN family peptides have been involved in the regulation of apoptosis, we explored whether HN modulates the propaoptotic effect of TNF-α in anterior pituitary cells. For this purpose, cultured anterior pituitary cells were incubated with TNF-α in presence of HN. The percentage of apoptotic cells was determined by TUNEL assay and lactotropes and somatotropes were identified by immunofluorescence. HN (2.5–10 µM) *per se* did not modify basal apoptosis of anterior pituitary cells (Fig. 6A–C). HN (2.5 µM) did not modify the proapoptotic action of TNF-α on anterior pituitary cells from OVX rats but at 5 and 10 µM concentrations abolished TNF-α-induced apoptosis and therefore 5 µM was the concentration chosen for all the following experiments. HN completely blocked as well the proapoptotic effect of TNF-α in lactotropes and somatotropes from OVX rats (Fig. 6D, E). HN exerted a similar antiapoptotic effect on total anterior pituitary cells, lactotropes and somatotropes from GNX male rats (Fig. 7A–C).

### Expression and action of HN family peptides in pituitary tumor cells

**Figure 8:**
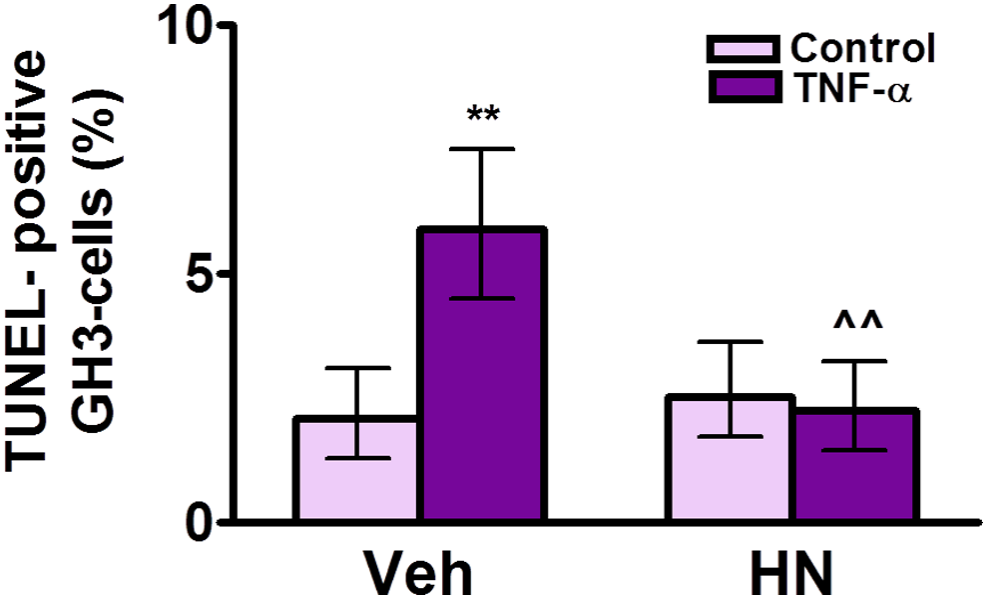
HN protects GH3 cells from TNF-α-induced apoptosis. Cultured GH3 cells were incubated with HN (5 µM) for 2 h before adding TNF-α (50 ng/ml) for 24 h. Apoptosis was assessed by the TUNEL method. Each column represents the percentage ± CL of TUNEL-positive cells (n≥1000 cells/group). Data from two separate experiments were analyzed by χ^2^ test. **p<0.01 respective control without TNF-α, ∧∧p<0.01 vs respective control without HN.

To explore whether HN plays a role in the apoptotic response of tumor pituitary cells we investigated HNr expression and HN activity in GH3 cells. FACS analysis of HNr expression revealed the presence of HNr in GH3 pituitary tumor cell line. 17β-estradiol (10^−9^ M) did not significantly affect the percentage of GH3 cells expressing HNr or HNr Gmean (Fig. S3). HN did not affect the basal percentage of TUNEL-positive GH3 cells but inhibited the apoptotic effect of TNF-α on GH3 cells (Fig. 8).

## Discussion

This study demonstrates for the first time that the HNr protein is present in the anterior pituitary gland, its expression showing sexual dimorphism. Our results also demonstrate that HN exerts antiapoptotic action on anterior pituitary cells, suggesting that this peptide could be involved in the homeostasis of this gland. The anterior pituitary gland undergoes considerable remodeling in several physiological conditions such as pregnancy, lactation and estrous cycle [21], [22]. Of the pituitary subpopulations, lactotropes and somatotropes have the highest cell turnover followed by corticotropes and gonadotropes [21]. We observed that HNr is expressed in both lactotropes and somatotropes, suggesting that HNr could participate in the regulation of cell renewal of these pituitary subpopulations.

Some studies have shown that the HN family peptides are present in hormone-dependent tissues such as testis [11], [14] and uterus [26]. Now, we detected HNr expression in anterior pituitary gland from both male and female rats. Little is known about regulatory mechanisms of HNr synthesis. It has been reported that HN content in plasma and hypothalamus decreases with age [7], [13]. Our study demonstrated that HNr expression in the anterior pituitary gland was lower in females than in males, suggesting that gonadal steroids are involved in the regulation of HNr expression in this gland. This difference in HNr expression could result from an inhibitory action of estrogens or, alternatively, from a stimulatory effect of androgens. We observed that ovariectomy increased HNr expression in the anterior pituitary gland, suggesting that ovarian steroids exert an inhibitory effect on HNr expression. In fact, *in vitro* estradiol decreased HNr expression in anterior pituitary cells from gonadectomized female and male rats, supporting the idea that estrogens are involved in the modulation of HNr expression *in vivo*, exerting direct inhibitory action. Estrogens are crucial determinants in the regulation of anterior pituitary function and maintenance of tissue homeostasis [27]. The changing pattern of circulating levels of estrogens during the estrous cycle modulates both anterior pituitary cell proliferation and death probably by modifying their sensitivity to proliferative and proapoptotic signals [28]. Therefore, inhibition of HNr expression by estradiol may have consequences on anterior pituitary cell turnover. Also, a fall of circulating in estrogen levels, and consequently a lack of estrogen feedback on the hypothalamus, can stimulate hypothalamic GnRH release, which was reported to increase HNr expression in immature rat testis [29]. Although it was not examined in the present study, inhibitory action of estrogens on HNr expression could possibly be exerted in other tissues.

A number of studies have demonstrated that HN suppresses cell death in neurons exposed to several insults such as β-amyloid [1], [30], oxidative stress [31], [32], serum deprivation [19] and hypoxia [33]. HN also has cytoprotective action in several cell types such as pancreatic β cells [20], lymphocytes [19] and testicular germ cells [16]. We previously reported that estradiol induces apoptosis of anterior pituitary cells from OVX rats not only by sensitizing them to proapoptotic stimuli such as dopamine [34], prolactin [35], TNF-α [36] and FasL [37] but also by direct proapoptotic action on lactotropes and somatotropes [38]. Our study demonstrated that HN prevented apoptosis caused by TNF-α in anterior pituitary cells, especially in lactotropes and somatotropes from both gonadectomized female and male rats. The expression of HNr in lactotropes and somatotropes and the antiapoptotic action of HN suggests a role of HNr in regulation of cell turnover of these pituitary subpopulations. Our results also showed that HNr is present and functional in GH3 cells, suggesting that HN could participate in the pathogenesis of pituitary tumors. Interestingly, the estrogenic modulation of HNr expression is absent in this tumor cell line.

HN has been shown to interact with two classes of receptors. It binds to the formylpeptide receptor-like-1 (FPRL-1) receptor [39], and also to a receptor complex with three subunits consisting of WSX-1 (IL-27 receptor), ciliary neurotrophic factor receptor α (CNTFR), and glycoprotein 130 (gp130), a subunit of interleukin-6 receptor [40], [41]. After binding to its specific receptor, HN mediates its protective effect through activation of STAT3 [42], JNK [43] and tyrosine kinases [42]. The three subunits described for the putative HN receptor, WSX-1, CNTFR, and gp130 were reported to be expressed in the anterior pituitary [44], [45]. Since HN family peptides can be released from cells [14], [18], HNr could exert its effects on anterior pituitary cells in an autocrine and/or paracrine manner. Another antiapoptotic mechanism has also been demonstrated by which intracellular HN interacts with proapoptotic Bcl-2 family members such as Bax [5], [29], BimEL [46] and tBid [47], their translocation to mitochondria prevailing and thereby inhibiting the formation of the apoptosome and activation of caspase-3. The mechanism by which HN protects anterior pituitary cells from proapoptotic stimuli, not examined in the present study, remains to be determined.

Since HN was reported to be overexpressed in gastric cancer cells and considering that HN has antiapoptotic activity in these cells, it was suggested that HN upregulation could be an important molecular event in tumorigenesis [48]. Apoptotic processes play an important role in maintaining the number of cells in the pituitary gland during different physiological events. Alterations in signaling pathways involved in the dynamic maintenance of pituitary cell populations have implications in tumorigenesis. Since HN exerts antiapoptotic action in normal and tumoral anterior pituitary cells, it is possible that changes in the expression of HN or its receptors may affect cell turnover, playing a role in the development of anterior pituitary tumors. Full elucidation of HN antiapoptotic action and its role in the physiology and pathology of the anterior pituitary gland will contribute to a better understanding of the basic mechanisms possibly involved in the pathogenesis of pituitary tumors.

## Supporting Information

Figure S1 An estrogen receptor antagonist reverses the inhibitory effect of estradiol on HNr expression in anterior pituitary cells from female rats. Cultured anterior pituitary cells from OVX rats were incubated with 17β-estradiol (E_2_, 10^−9^ M) or vehicle (Veh, ethanol 1 µl/l) in the presence of ICI 182 780 (ICI, 10^−7^ M) for 24 h, immunostained for HNr and analyzed by flow cytometry. Each column represents the mean ± SE of the percentage of HNr-positive cells from 2 independent experiments (4 replicates each one). **p<0.01 vs respective control without estradiol, ∧p<0.05 vs respective control without ICI. Two-way ANOVA followed by planned comparisons.
(TIF)

Figure S2 Gonadectomy does not modify HNr expression in anterior pituitary from male rats. Dispersed anterior pituitary cells from SHAM and GNX rats were immunostained for HNr and analyzed by flow cytometry. Each column represents the mean ± SE (n = 4 animals per group) of (A) the percentage of HNr-positive cells, and (B) the fluorescence intensity of HNr staining (Gmean). The upper panels show representative dot plots and histograms of HNr expression in anterior pituitary cells from SHAM and GNX rats. NS, Student’s *t* test.
(TIF)

Figure S3 Estradiol does not modify HNr expression in GH3 cells. Cultured GH3 cells were incubated with 17β-estradiol (E_2_, 10^−9^ M) or vehicle (Veh, ethanol 1 µl/l) for 24 h, immunostained for HNr and analyzed by flow cytometry. Each column represents the mean ± SE from 3 independent experiments (3 replicates each) of (A) the percentage of HNr-positive cells, and (B) the fluorescence intensity of HNr staining (Gmean). The upper panels show representative dot plots and histograms of HNr expression in GH3 cells. NS, Student’s *t* test.
(TIF)
