# Correction: Antiapoptotic Factor Humanin Is Expressed in Normal and Tumoral Pituitary Cells and Protects Them from TNF-α-Induced Apoptosis

**DOI:** 10.1371/journal.pone.0124589

**Published:** 2015-04-10

**Authors:** María Florencia Gottardo, Gabriela Jaita, María Laura Magri, Sandra Zárate, Mariela Moreno Ayala, Jimena Ferraris, Guadalupe Eijo, Daniel Pisera, Marianela Candolfi, Adriana Seilicovich

The concentrations of Humanin appear incorrectly throughout the article. The concentrations of Humanin should be 0.25, 0.5 and 1 μM instead of 2.5, 5 and 10 μM, respectively. Please see below for details regarding the locations of the errors in the article.

The eighth sentence of the Abstract should read: HN (0.5 μM) *per se* did not modify basal apoptosis of anterior pituitary cells but completely blocked the proapoptotic effect of TNF-α in total anterior pituitary cells, lactotropes and somatotropes from both female and male rats.

The last sentence of the Materials and Methods subsection titled “Cell Culture” should read: To determine apoptosis, cells were further preincubated with HN (0.25–1 μM) for 2 h before adding TNF-α (50 ng/ml) for 24 h in the same medium with or without E_2_ or DHT.

The fifth and sixth sentences of the Results subsection titled “HN blocked TNF-α-induced apoptosis of anterior pituitary cells” should read: HN (0.25–1 μM) *per se* did not modify basal apoptosis of anterior pituitary cells (Fig 6A–6C). HN (0.25 μM) did not modify the proapoptotic action of TNF-α on anterior pituitary cells from OVX rats but at 0.5 and 1 μM concentrations abolished TNF-α-induced apoptosis and therefore 0.5 μM was the concentration chosen for all the following experiments.

The complete, correct Fig 6 legend is:

**Fig 6:**
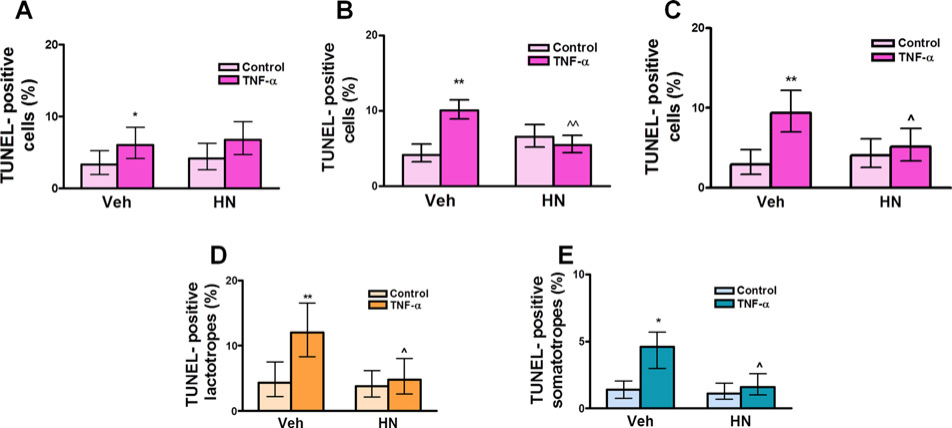
HN protects anterior pituitary cells from TNF-α-induced apoptosis in female rats. Cultured anterior pituitary cells from OVX rats were incubated with 17β-estradiol (E_2_, 10^-9^ M) for 24 h and then with HN 0.25 μM (A), 0.5 μM (B), or 1 μM (C) for 2 h before adding TNF-α (50 ng/ml) for an additional 24 h. Apoptosis was assessed by the TUNEL method. In cells incubated with 0.5 μM HN, apoptotic lactotropes (D) and somatotropes (E) were identified by immunofluorescence. Each column represents the percentage ± CL of TUNEL-positive anterior pituitary cells (A, B, C, n≥1500 cells/group), lactotropes (D, n≥1600 cells/group) or somatotropes (E, n≥1800 cells/group). Data from at least two separate experiments were analyzed by χ^2^ test. *p<0.05, **p<0.01 vs respective control without TNF-α; ∧p<0.05, ∧∧p<0.01 vs respective control without HN.

The complete, correct Fig 7 legend is:

**Fig 7:**
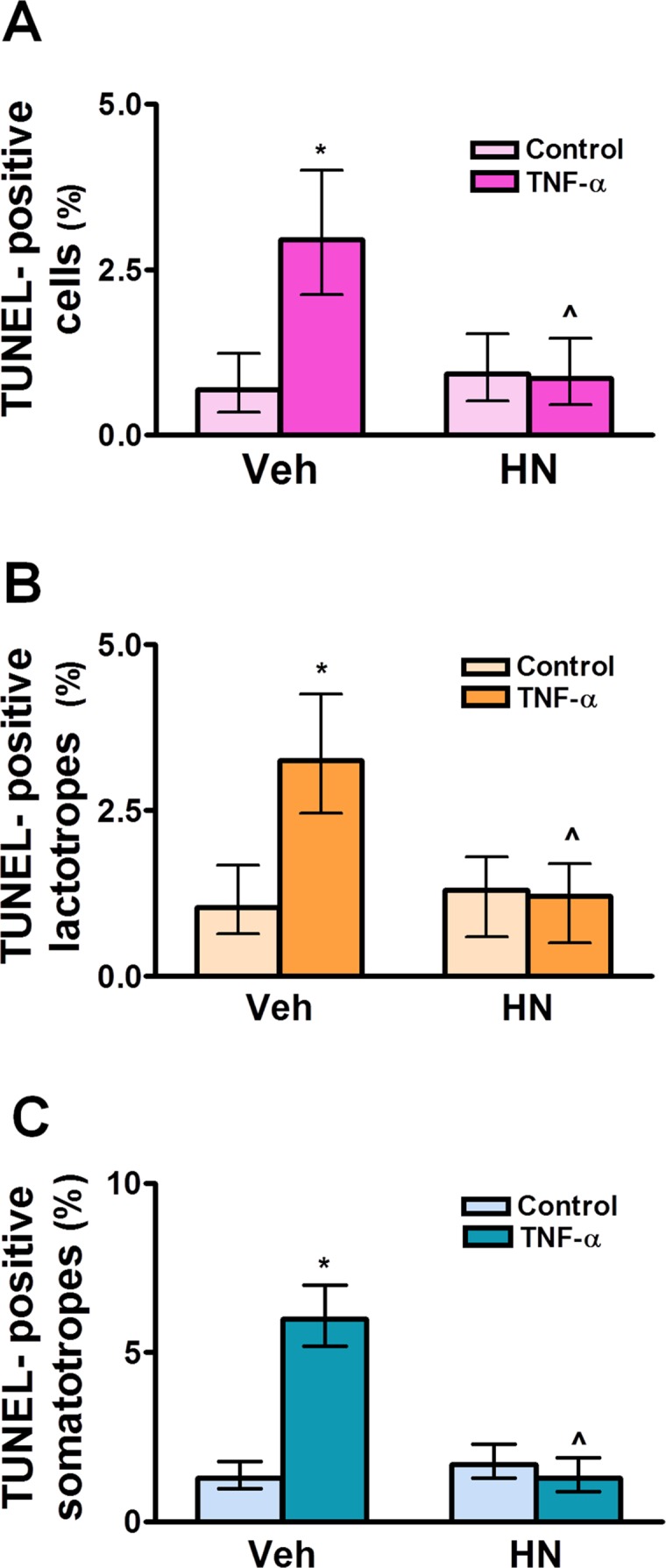
HN protects anterior pituitary cells from TNF-α-induced apoptosis in male rats. Cultured anterior pituitary cells from GNX male rats were incubated with dihydrotestosterone (DHT, 10^-8^ M) for 24 h and then with HN (0.5 μM) for 2 h before adding TNF-α (50 ng/ml) for an additional 24 h. Apoptosis was assessed by the TUNEL method and lactotropes and somatotropes detected by immunofluorescence. Each column represents the percentage ± CL of TUNEL-positive anterior pituitary cells (A, n≥1400 cells/group), lactotropes (B, n≥1400 cells/group) or somatotropes (C, n≥1300 cells/group). Data from at least two separate experiments were analyzed by χ^2^ test. *p<0.05 respective control without TNF-α; ∧p<0.05 vs respective control without HN.

The complete, correct Fig 8 legend is:

**Fig 8:**
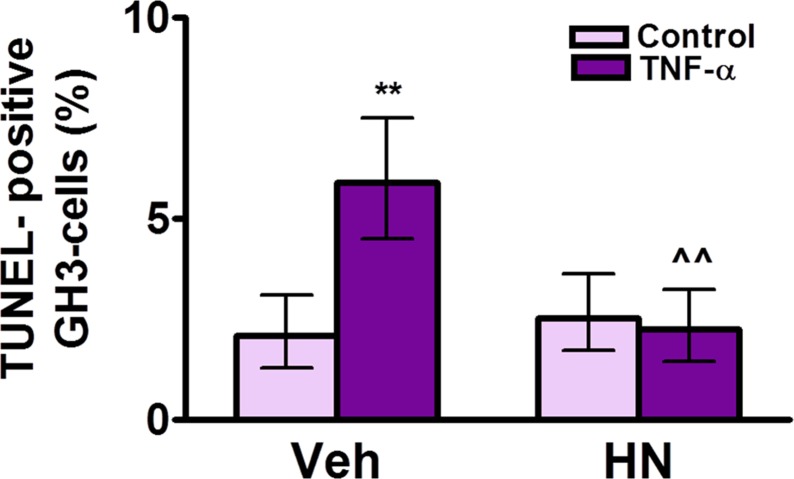
HN protects GH3 cells from TNF-α-induced apoptosis. Cultured GH3 cells were incubated with HN (0.5 μM) for 2 h before adding TNF-α (50 ng/ml) for 24 h. Apoptosis was assessed by the TUNEL method. Each column represents the percentage ± CL of TUNEL-positive cells (n≥1000 cells/group). Data from two separate experiments were analyzed by χ^2^ test. **p<0.01 respective control without TNF-α, ∧∧p<0.01 vs respective control without HN.
