# Supplementary figures and images for: Antiapoptotic Factor Humanin Is Expressed in Normal and Tumoral Pituitary Cells and Protects Them from TNF-α-Induced Apoptosis
Source: PLoS One. 2014 Oct 31;9(10):e111548. doi: 10.1371/journal.pone.0111548 (PMC4216097; doi:10.1371/journal.pone.0111548)

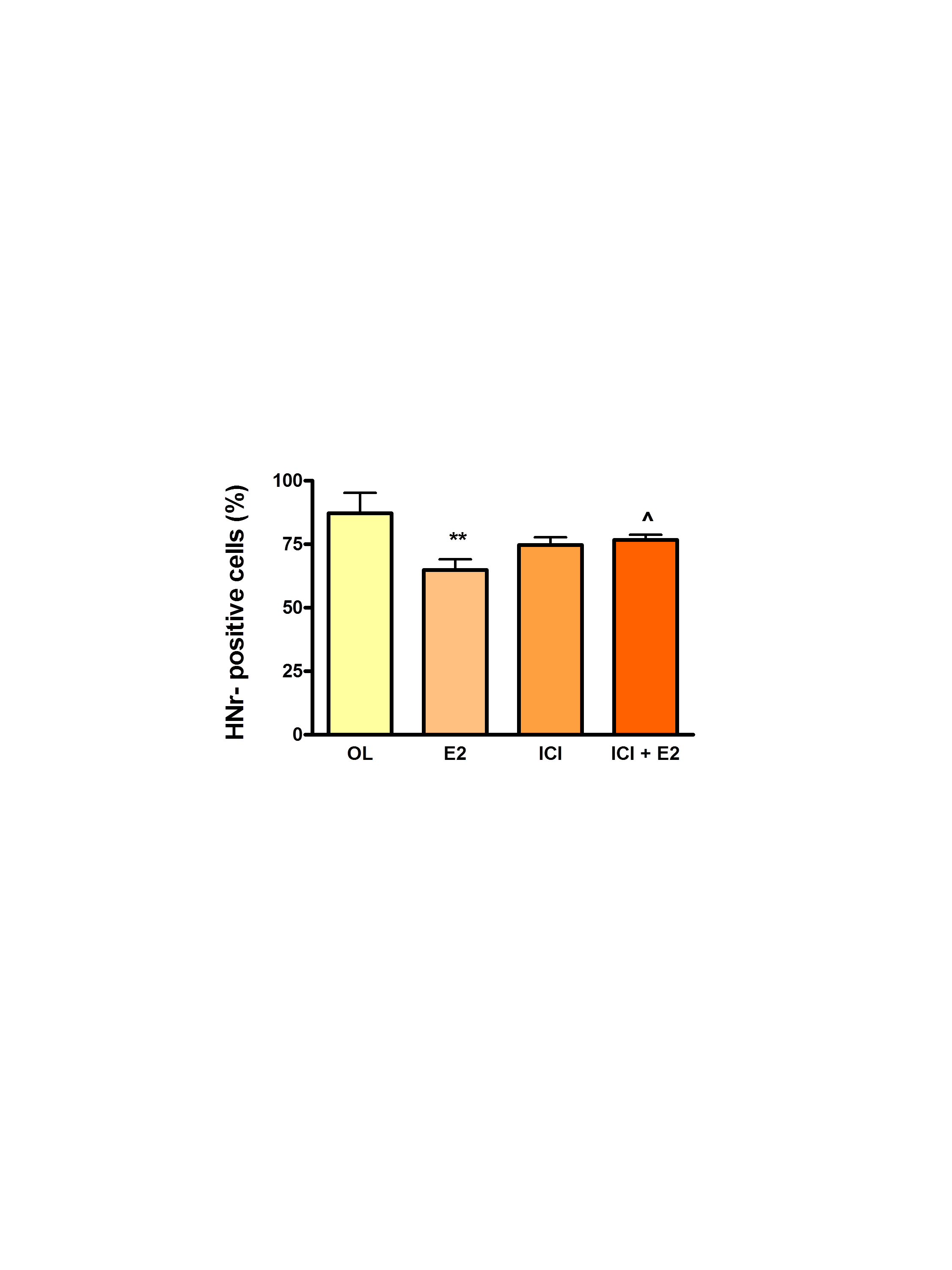

Supplement: Figure S1 — An estrogen receptor antagonist reverses the inhibitory effect of estradiol on HNr expression in anterior pituitary cells from female rats. Cultured anterior pituitary cells from OVX rats were incubated with 17β-estradiol (E2, 10−9 M) or vehicle (Veh, ethanol 1 µl/l) in the presence of ICI 182 780 (ICI, 10−7 M) for 24 h, immunostained for HNr and analyzed by flow cytometry. Each column represents the mean ± SE of the percentage of HNr-positive cells from 2 independent experiments (4 replicates each one). **p<0.01 vs respective control without estradiol, ∧p<0.05 vs respective control without ICI. Two-way ANOVA followed by planned comparisons. (TIF) [file pone.0111548.s001.tif]

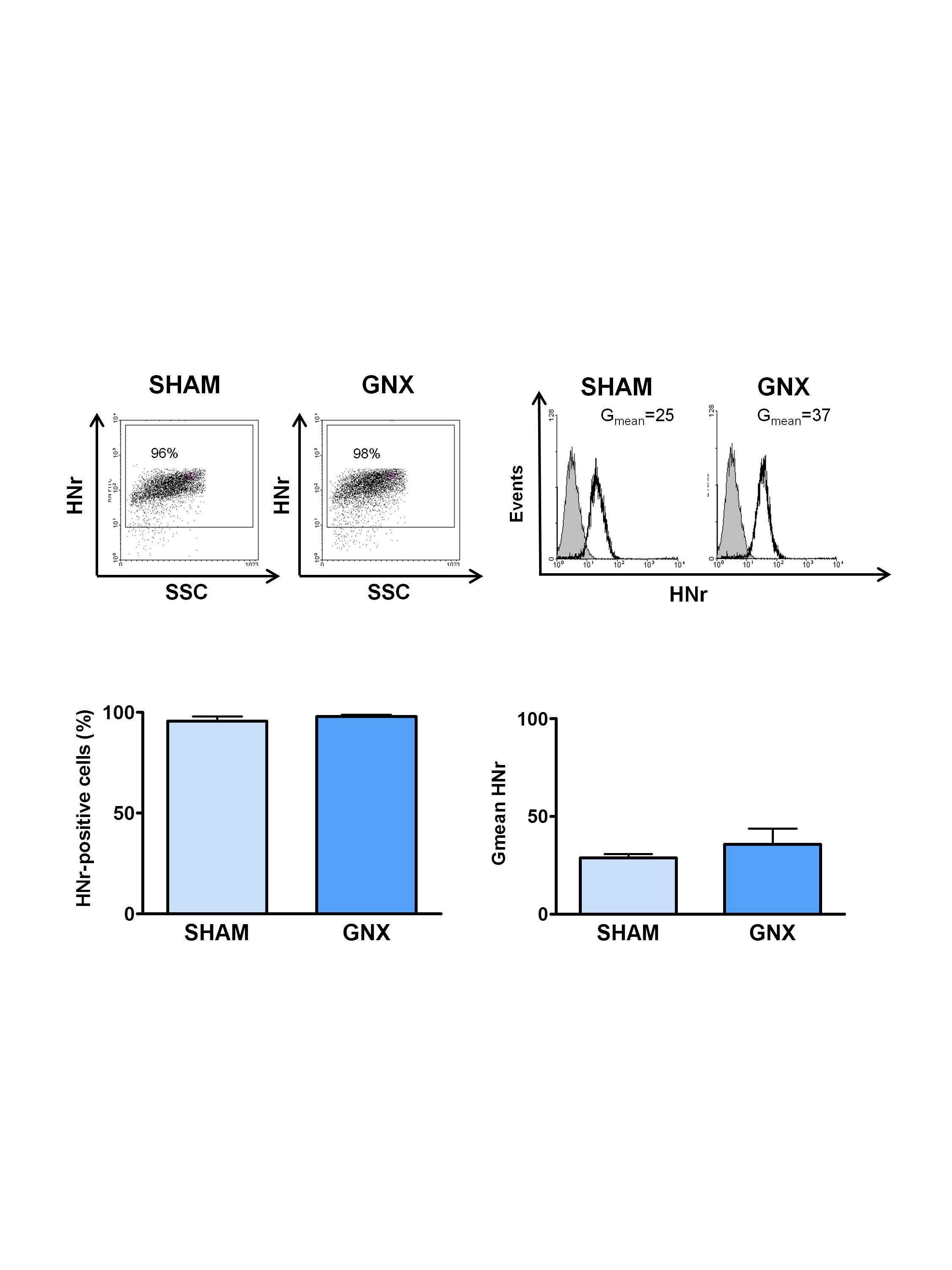

Supplement: Figure S2 — Gonadectomy does not modify HNr expression in anterior pituitary from male rats. Dispersed anterior pituitary cells from SHAM and GNX rats were immunostained for HNr and analyzed by flow cytometry. Each column represents the mean ± SE (n = 4 animals per group) of (A) the percentage of HNr-positive cells, and (B) the fluorescence intensity of HNr staining (Gmean). The upper panels show representative dot plots and histograms of HNr expression in anterior pituitary cells from SHAM and GNX rats. NS, Student’s t test. (TIF) [file pone.0111548.s002.tif]

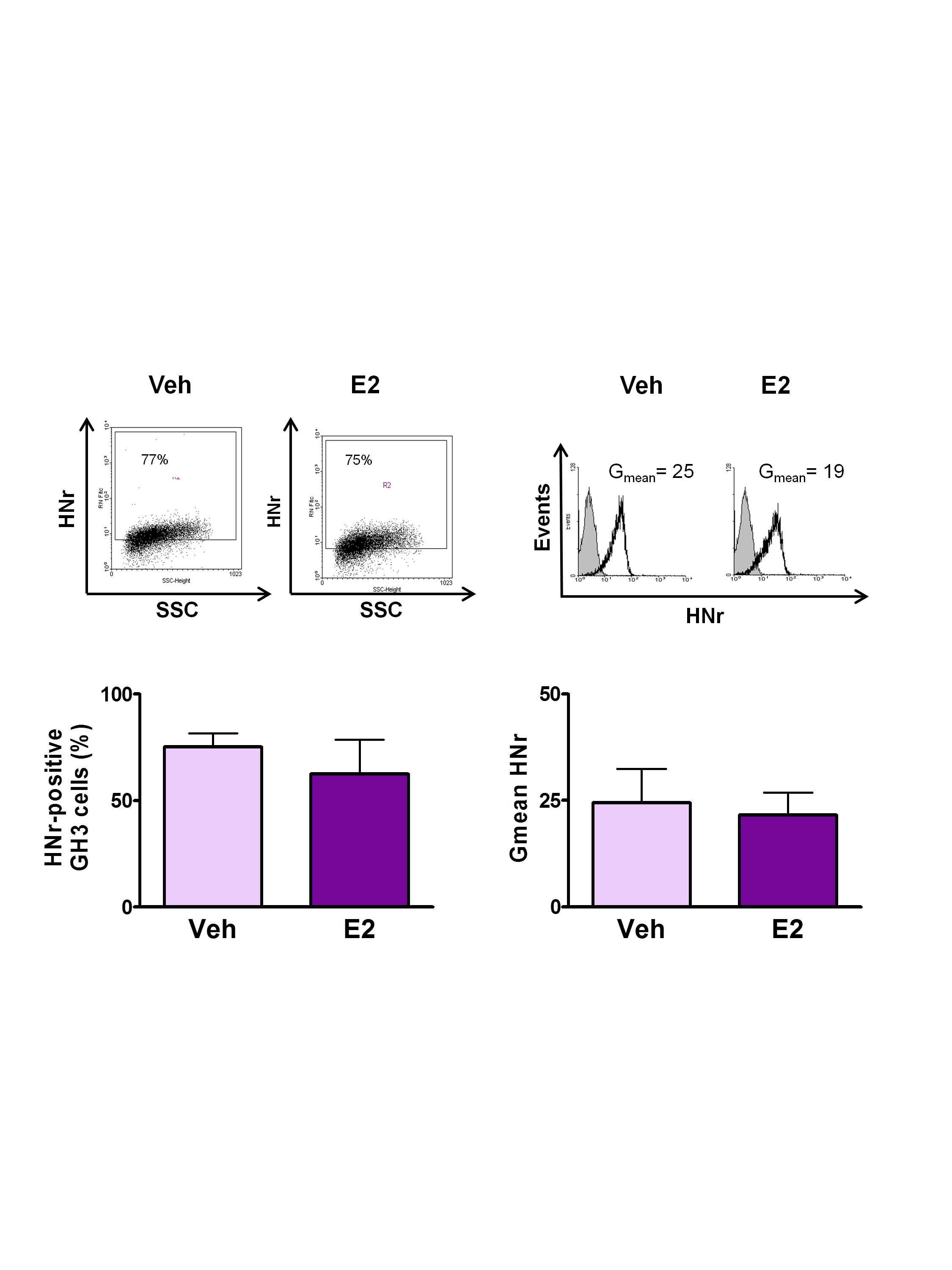

Supplement: Figure S3 — Estradiol does not modify HNr expression in GH3 cells. Cultured GH3 cells were incubated with 17β-estradiol (E2, 10−9 M) or vehicle (Veh, ethanol 1 µl/l) for 24 h, immunostained for HNr and analyzed by flow cytometry. Each column represents the mean ± SE from 3 independent experiments (3 replicates each) of (A) the percentage of HNr-positive cells, and (B) the fluorescence intensity of HNr staining (Gmean). The upper panels show representative dot plots and histograms of HNr expression in GH3 cells. NS, Student’s t test. (TIF) [file pone.0111548.s003.tif]
